# Supplementary material for: Resolving the full spectrum of human genome variation using Linked-Reads
Source: Genome Res. 2019 Apr;29(4):635–45. doi: 10.1101/gr.234443.118 (PMC6442396; doi:10.1101/gr.234443.118)
Supplement: Supplemental Material [file supp_gr.234443.118_Supplemental_longranger-2.2.zip › longranger-2.2.tar.bz2!/lr2.2-public/lib/go/src/code.google.com/vitess/vitess.io/_includes/latest-posts-grid.html]

### Latest {{ page.categories | first | replace: '-',' ' | capitalize }}

{% capture category\_name %}{{ page.categories | first }}{% endcapture %}
{% for post in site.categories.[category\_name] limit:4 %}
{% endfor %}
